# Analysis of the Implementation of Telehealth Visits for Care of Patients With Cancer in Houston During the COVID-19 Pandemic

**DOI:** 10.1200/OP.20.00572

**Published:** 2020-10-07

**Authors:** Jorge G. Darcourt, Kalia Aparicio, Phillip M. Dorsey, Joe E. Ensor, Eva M. Zsigmond, Stephen T. Wong, Chika F. Ezeana, Mamta Puppala, Kirk E. Heyne, Charles E. Geyer, Robert A. Phillips, Roberta L. Schwartz, Jenny C. Chang

**Affiliations:** ^1^ Houston Methodist Hospital, Houston, TX; ^2^ Houston Methodist Hospital Research Institute, Houston, TX; ^3^ Systems Medicine and Bioengineering, Houston Methodist Cancer Center, Houston, TX

## Abstract

The purpose of this study was to evaluate the use of telemedicine amid the SARS-CoV-2 pandemic in patients with cancer and assess barriers to its implementation.

**PATIENTS AND METHODS:**

Telehealth video visits, using the Houston Methodist MyChart platform, were offered to patients with cancer as an alternative to in-person visits. Reasons given by patients who declined to use video visits were documented, and demographic information was collected from all patients. Surveys were used to assess the levels of satisfaction of treating physicians and patients who agreed to video visits.

**RESULTS:**

Of 1,762 patients with cancer who were offered telehealth video visits, 1,477 (83.8%) participated. The patients who declined participation were older (67.7 *v* 60.2 years; *P* < .0001), lived in significantly lower-income areas (*P* = .0021), and were less likely to have commercial insurance (*P* < .0001) than patients who participated. Most participating patients (92.6%) were satisfied with telehealth video visits. A majority of physicians (65.2%) were also satisfied with its use, and 74% indicated that they would likely use telemedicine in the future. Primary concerns that physicians had in using this technology were inadequate patient interactions and acquisition of medical data, increased potential for missing significant clinical findings, decreased quality of care, and potential medical liability.

**CONCLUSION:**

Oncology/hematology patients and their physicians expressed high levels of satisfaction with the use of telehealth video visits. Despite recent advances in technology, there are still opportunities to improve the equal implementation of telemedicine for the medical care of vulnerable older, low-income, and underinsured patient populations.

## INTRODUCTION

On March 11, 2020, the WHO declared the COVID-19 outbreak a global pandemic,^1^ and as a result, regular patient care was affected worldwide. For hematology and oncology patients, this has been specially challenging because these patients are predominantly of older age, are usually male, and have comorbidities that are associated with adverse outcomes from SARS-CoV-2 infection.^2,3^ Other reports have shown that patients with cancer are more likely to be intubated,^4^ have a higher incidence of adverse events,^5,6^ and have a higher odds ratio of increased complications.^7^

Telemedicine has rapidly evolved as a solution to continued patient care during the COVID-19 pandemic, especially for hematology and oncology patients. In recent years, efforts to effectively incorporate video telehealth visits into regular patient care were impeded by regulatory and reimbursement problems, available technology, and lack of familiarity of both providers and patients with the system. The COVID-19 pandemic and the paramount importance of social distancing, together with regulatory changes in Centers for Medicare and Medicaid Services (CMS) reimbursement, have resulted in the elimination of some of these barriers to safe and effective patient care via telemedicine.

Prior publications have highlighted barriers to adopting telemedicine worldwide. A systematic review of the challenges associated with the use of telemedicine identified the following primary deterrents to implementation: technically untrained staff (11%), resistance to change by the medical providers (8%), cost (8%), reimbursement (5%), and age of patients, which is related to a lack of exposure and training in new technologies (5%).^8,9^ A recent review of patients with cancer identified technology costs, inconsistent billing, reimbursement regulations, and data security risks as major barriers to dissemination. Another important consideration in the use of telemedicine is the digital divide that hampers equal access to these types of health care services.^10^ Investments in the information technology infrastructure^11^ and liberalization of the Medicare policies that relate to telehealth are key factors for the successful navigation of the current health care crisis posed by the COVID-19 pandemic.^12^

The objective of this prospective observational study was to assess the effectiveness of using telemedicine in the treatment of patients with cancer during the SARS-CoV-2 pandemic and identify barriers to the implementation of this new technology for patient care. At the inception of televisits, we documented the reasons for declining telemedicine video visits across our seven cancer centers. We analyzed the demographics and characteristics of the patients who accepted and who declined to have video visits. We report on these differences to further understand and advance telemedicine care for malignant hematology and oncology patients.

## PATIENTS AND METHODS

### Population

Before COVID-19, of the approximately 40,000 ambulatory visits per year to the Houston Methodist Cancer Center (HMCC), virtually 100% were in-person visits. At the onset of the SARS-CoV-2 pandemic in Houston in March 2020, as an alternative to in-person visits, approximately 50% of our patients were offered telehealth video visits using the Houston Methodist MyChart video platform from the seven Houston Methodist cancer centers across the greater Houston area (GHA). MyChart video visits are secure, Health Insurance Portability and Accountability Act–compliant virtual visits that occur in Epic and MyChart between a patient and his or her provider. We used Vidyo integration for the video component. The patients who agreed to have a MyChart video visit were instructed to download the application on their smart devices (telephone or tablet) by a medical or front desk assistant who was also readily available to troubleshoot the medical visit at the time of the appointment. The Houston Methodist Institutional Review Board reviewed the study and granted a waiver as a hospital operations/quality improvement study. Three data sets were prospectively collected: data set 1, patients who preferred not to or could not pursue a MyChart video visit were asked by telephone about the reasons for declining, and their answers were recorded weekly across the system; data set 2, patient postvideo survey using a Microsoft Poll questionnaire was created based on the Telehealth Satisfaction Scale^8^ and was sent by e-mail to the patients who agreed to have a MyChart video visit; and data set 3, physician survey (Microsoft Poll questionnaire) was sent to our 23 hematologists and medical oncologists after the implementation of the telehealth video visits, on week 3. Patients received two reminders to complete the surveys.

### Statistical Analysis

Univariable analysis was conducted on the characteristics of both groups: group 1, patients who were able to use MyChart video visits (accepted); and group 2, patients who could not use the platform (declined). We described their characteristics in terms of mean age, sex, race/ethnicity, median income based on zip code data, and type of insurance (commercial *v* Medicare/Medicaid) based on anonymous medical record reviews. Multivariable logistic regression analysis was performed to compare the differences in implementation between the patient populations. For continuous factors, such as age and median income, differences between telemedicine participants and nonparticipants were assessed using a two-sided pooled or Satterthwaite *t* test as appropriate. A folded F test was used to assess variance heterogeneity. Normality was assessed objectively using a Shapiro-Wilk test and assessed visually by inspecting normal probability Q-Q plots. If the data suggested a departure from normality, a Wilcoxon rank-sum test was used to investigate differences between the two cohorts. For categorical factors, such as sex and race, a χ^2^ or Fisher’s exact test was used to evaluate cohort differences. Univariable and multivariable logistic regression was used to determine the predictive significance of each factor. All analyses were conducted using SAS software (version 9.4; SAS Institute, Cary, NC). Statistical significance was defined as *P* < .05.

## RESULTS

### Patient Characteristics

**FIG 1.**
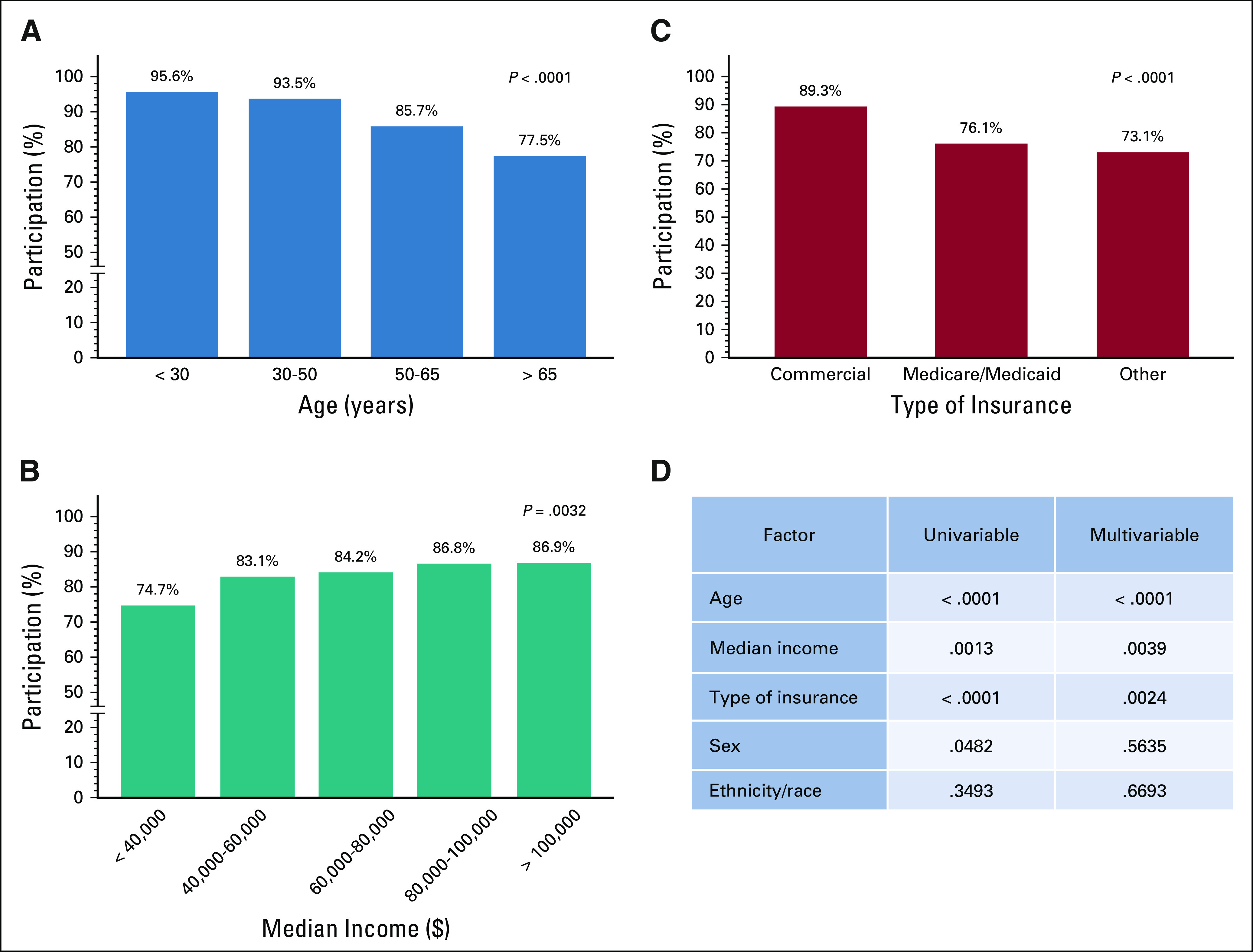
In univariable and multivariable analyses, (A, D) younger age, (B, D) higher median income, and (C, D) having commercial insurance were associated with participation in telemedicine video visits. (D) In univariable analysis, female sex was associated with participation, but this association was not statistically significant in multivariable analysis. Age and median income are depicted as continuous variables and type of insurance as categorical.

From March 16 to April 20, 2020, of the 1,762 patients who were offered MyChart video visits, 1,477 (83.8%) accepted and 285 (16.2%) declined. By univariable analysis, there was a statistically significant difference between patients who accepted video visits versus those who declined, in terms of younger mean age (60.2 *v* 67.7 years; *P* < .0001), higher median annual income ($72,300 *v* $66,800; *P* = .0013), private insurance (commercial *v* Medicare/Medicaid/other; *P* < .0001), and female sex (female *v* male; *P* < .0482). There was no significant participation difference in terms of race/ethnicity (*P* = .3493; Table 1). Next, multivariable analysis was used to evaluate the socioeconomic determinants of patients agreeing to participate versus those declining to participate in video visits. Independent variables were mean age, median income, and type of insurance. There was a significant difference between the mean age of the two cohorts (*P* < .0001). The patients who declined to participate in MyChart video visits were significantly older, and a logistic regression model predicting participation based on age exhibited a significant effect based on age. Additionally, the median income for the patient zip code was known for 1,743 of the 1,762 patients. There was a significant difference in mean income between the two cohorts (*P* = .0013), with patients in lower-income areas declining MyChart video visits. A logistic regression model predicting participation based on median income ($1,000) exhibited a significant effect. Furthermore, the type of insurance was known for 1,747 of the 1,762 patients. χ^2^ analysis showed a significant difference in the distribution of the type of insurance used by participants versus nonparticipants (*P* < .0001), where patients with commercial insurance were more likely to participate (Fig 1).

**TABLE 1.**
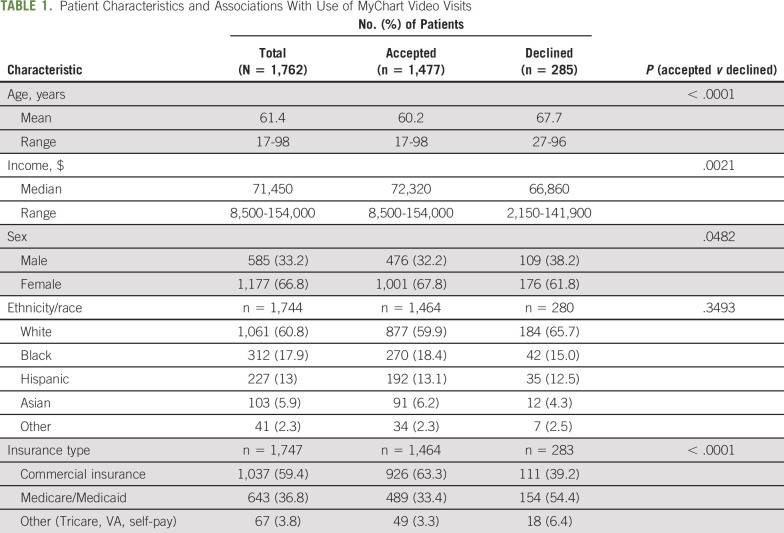
Patient Characteristics and Associations With Use of MyChart Video Visits

### Patient Survey Results

**FIG 2.**
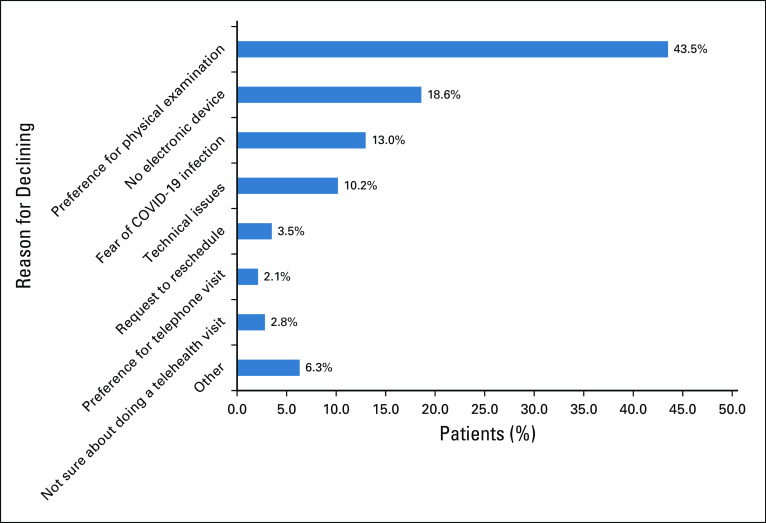
Reasons for declining telehealth visits.

Among the 285 patients (16.2%) who declined a video visit through MyChart, the main reasons included preference for a face-to-face visit (43.5%) and technologic problems (28.8%). The latter resulted from lack of Internet or mobile device (18.6%) or technical issues with the MyChart application (10.2%). Some other patients cited the need to reschedule for a future date because of fear of COVID-19 infection (13.0%) or preference for rescheduling with no particular reason (3.5%), preference for a telephone call (2.1%), being unsure about doing a telehealth visit (2.8%), and other unspecified reasons (6.3%; Fig 2).

A patient follow-up survey was conducted to examine patient satisfaction with video visits for the patients who had agreed to participate in MyChart video visits within 3 weeks of their appointment. Of these patients, 21% completed the surveys. The responders were more likely women (73.5%) rather than men (26.2%). The age distribution of patients who responded were as follows: age > 60 years (63.9%), between 45 and 60 years (24.9%), between 30 and 44 years (8.9%), and between 18 and 29 years (0.6%). These patients were seen by their physicians at our main academic campus hospital (25.6%) or one of the six regional cancer centers in the GHA (74.6%).

Satisfaction with the overall quality of the video visit was surveyed. Most patients who completed the survey were satisfied (92.6%), and 83.4% of these were very satisfied. Regarding the ease of use of the MyChart video visit, 91.7% of patients were satisfied, and 76.7% of these patients did not require any technical support. For patients who required technical support, 72% used the help of a Houston Methodist employee, and 53% were helped by a family member or friend. The quality of interaction with their physician and the ability of the physician to address their clinical questions on the MyChart video visit were reported as satisfactory by 96.7% and 96.8% of patients, respectively. Most patients (97.1%) were also satisfied with their sense of privacy during the visits, and these patients indicated that they would be highly likely (73.2%) or somewhat likely (17.2%) to choose another MyChart video visit in the future. For future visits, the patients responded that they would like all (18.8%), most (36.4%), some (31.2%), few (8.9%), or none (3.8%) of their future visits to occur via MyChart video.

### Physician Survey Results

A physician survey was sent to our 23 hematologists and medical oncologists (academic, 48%; community, 52%) 3 weeks after we started to provide MyChart video visits, with 100% response. Of the 23 hematologists and medical oncologists, 20 physicians (87%) were age < 60 years. The survey revealed that 91% of the physicians used MyChart video visits to assess patients, and 87% of these were using the MyChart video visits to see new patients for hematology or oncology appointments. In addition, 52.5% of the physicians were seeing > 50% of their patients using virtual video visits, and 87% of physicians used it for > 25% of their patient visits.

**FIG 3.**
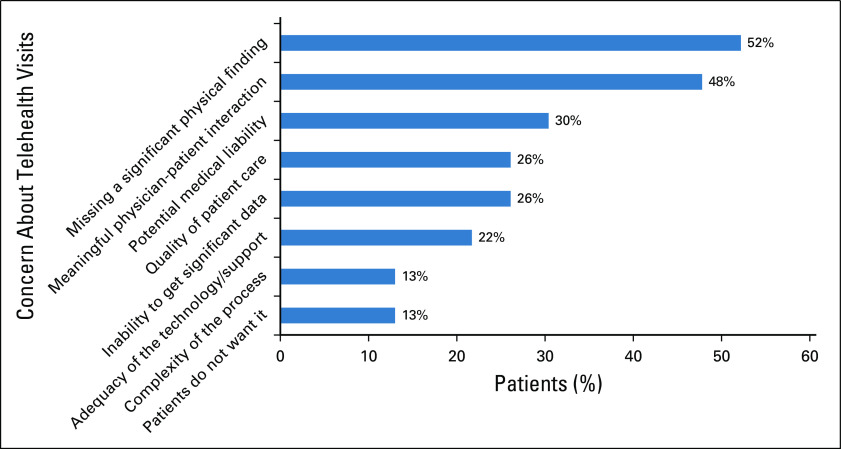
Physician survey: concerns about telehealth visits.

Physicians’ concerns included: fear of missing significant clinical findings (52.2%), lack of meaningful physician-patient interaction (47.8%), decrease in quality of care (26.1%), inability to get adequate data/information (26%), potential medical liability (30.4%), complexity of the process (13%), inadequate technologic support (21.7%). and patients not wanting video visits (13%; Fig 3).

Of these physicians, 65.2% were satisfied or very satisfied with MyChart video visits, whereas 21.7% were neutral and 13.1% were dissatisfied or very dissatisfied with the system. Physicians responded that they were highly likely (13%), somewhat likely (17.4%), neutral (17.4%), unlikely (39.1%), or very unlikely (13%) to continue using MyChart video visits for new patients after the COVID-19 crisis. In contrast, they reported that they were highly likely (43.5%), somewhat likely (30.5%), neutral (13%), unlikely (8.7%), or very unlikely (4.3%) to continue using MyChart video visits for established patients after the COVID-19 crisis. Finally, 52.2% of the treating physicians felt that approximately 26% to 50% of their patients would prefer MyChart video visits instead of some in-person follow-up visits; 30.5% felt that 0% to 25% of their patients would likely continue this method of visits, and 17.4% felt that > 50% of their patients would prefer to use some MyChart video visits in the future. Of the physicians surveyed, 65.2% were highly likely or likely to recommend the use of MyChart video visits to their colleagues for patient care.

## DISCUSSION

In this prospective study, we report that patients age ≥ 60 years successfully used telemedicine, highlighting the increased adoption of technology platforms by older patient populations. There was, however, a statistically significant difference in the mean age of the patients who accepted MyChart video visits compared with those who declined its use. More than 90% of the patients who accepted the use of MyChart video visits expressed a high level of satisfaction and noted the ease of use of this technology. Most patients expressed a desire to continue with such visits, because they felt that their questions were answered, and they had received adequate medical care. These findings emphasize that even though great advances have been made in increasing acceptance, lowering the cost, and improving portability and ease of use of technology as a tool for patient care, the older population is still at a disadvantage. Additional efforts must be made to reach these patients who often present with comorbidities and often live by themselves and lack additional technologic support.

If these findings are replicated in other studies, video telehealth visits will also be an important future strategy for specialty cancer care. Such an approach could decrease the disparities in health care delivery to rural and distant locations and for patients with transportation issues. Our successful experience enrolling most of our patients in MyChart video visits was accomplished by the efforts of the support staff at HMCC, and it highlights the importance of having adequately trained and readily available personnel to help patients. A lower median income and lack of commercial insurance were also negatively associated with the ability of patients to use telemedicine video visits. No differences resulting from race/ethnicity in the adoption of the technology were observed. The sex difference in favor of use by female patients was erased on multivariable analysis because of an older male population. Our survey results show a disproportionate number of female responders, but this aligns with our current hematology and oncology practice, which is heavily weighted toward caring for female patients.

Most of our physicians were comfortable taking care of their patients using video visits, although they voiced concerns about the quality of care or missing important data because of a lack of physical examination. Also, patients seemed to be more enthusiastic about having future appointments using telemedicine video visits (all, 18.8%; most, 36%) than the physician group, where only 17.4% of responders felt that > 50% of their patients would like to use MyChart video visits for some of their future visits. Physicians seemed to be more concerned about aspects inherent to physician-patient interaction and quality of care rather than technologic or reimbursement-related issues. A key problem for medical oncology and hematology practice is the paramount importance of the physical examination, including palpation of tumors, lymph nodes, liver, and spleen, along with breast examination. Multiple tools are being developed to correct these concerns in the form of smart devices and sonograms that can be adapted for remote clinical use. Monitoring of ECG (KardiaMobile), as well as weight and blood pressure, using devices such as AppleHealth/GoogleFit, Withings Smart Devices, Tytocare Remote Exam Kit, and HexoSkin represents a rapidly evolving solution to fulfill these needs.

Of note, > 75% of our patients who responded to this survey belonged to the GHA, an area that encompasses 10,000 square miles of our catchment area. This highlights the suitability of these types of services in both remote areas as well as densely populated metropolitan areas that rely on a main academic center for high-complexity cases. Other innovative initiatives include the Smart Health Stations from Higi, allowing patients to share their health information with their medical providers using health kiosks placed in nontraditional community locations such as churches, community centers, supermarkets, and schools.

The Houston Methodist Hospital system was able to rapidly adopt telemedicine for the treatment of patients with cancer, a highly vulnerable population. In the future, the willingness of physicians and medical organizations to continue the use of these technologic platforms will be affected by the capability of maintaining equal reimbursement for the provided services. This will be equally important for the implementation of telemedicine technologies as a strategy to better serve remote and underserved areas that are in desperate need of high-quality specialty care closer to home.

Limitations of the study include the lack of external validation of the results, because the study was conducted at a single institution, using one specific telehealth platform over a short, 6-week study period, and the possibility of selection bias by physicians who offered telehealth video visits to their patients at their own discretion.

In conclusion, the COVID-19 crisis has accelerated the rate of telehealth use as part of our armamentarium for patient care. Key aspects of the success of video telehealth visits have been the willingness of the CMS and insurance companies to reimburse practitioners and hospital practices as well as the level of readiness and integration of the electronic health record and information technology systems. In this study, we report the feasibility of using telemedicine for the care of patients with cancer. Because patients are critical players in this consumer-driven market, their favorable assessment of telehealth visits may drive our medical practices and lawmakers toward making this type of visit a permanent part of patient care, even in the aftermath of the current pandemic. Telemedicine, with further development of supportive devices, applications, and other technologies, has the potential to enhance clinical interactions between patients and physicians, without diminishing the quality of care. The current health crisis will accelerate the needed improvements in patients’ access to care in vulnerable populations, such as those with cancer, the elderly, and patients with lower economic income. Implementation of more accessible telehealth visits may also decrease the health disparities observed between rural and urban communities and facilitate the care of patients who have transportation restrictions or who need specialized medical assessments. We believe that the COVID-19 pandemic is presenting us with a unique opportunity to expand the use of telemedicine as a critical part of state-of-the-art medical care.
